# Reducing the number of unnecessary biopsies for mammographic BI-RADS 4 lesions through a deep transfer learning method

**DOI:** 10.1186/s12880-023-01023-4

**Published:** 2023-06-13

**Authors:** Mingzhu Meng, Hong Li, Ming Zhang, Guangyuan He, Long Wang, Dong Shen

**Affiliations:** 1 Department of Radiology, The Affiliated Changzhou No 2 People’s Hospital of Nanjing Medical University, Changzhou, 213164 Jiangsu Province P. R. China; 2 Department of Radiology, The Second Affiliated Hospital of Soochow University, Suzhou, 215004 Jiangsu Province P.R. China

**Keywords:** Residents, Deep transfer learning, Fine-tuning, Mammography, Breast lesions

## Abstract

**Background:**

In clinical practice, reducing unnecessary biopsies for mammographic BI-RADS 4 lesions is crucial. The objective of this study was to explore the potential value of deep transfer learning (DTL) based on the different fine-tuning strategies for Inception V3 to reduce the number of unnecessary biopsies that residents need to perform for mammographic BI-RADS 4 lesions.

**Methods:**

A total of 1980 patients with breast lesions were included, including 1473 benign lesions (185 women with bilateral breast lesions), and 692 malignant lesions collected and confirmed by clinical pathology or biopsy. The breast mammography images were randomly divided into three subsets, a training set, testing set, and validation set 1, at a ratio of 8:1:1. We constructed a DTL model for the classification of breast lesions based on Inception V3 and attempted to improve its performance with 11 fine-tuning strategies. The mammography images from 362 patients with pathologically confirmed BI-RADS 4 breast lesions were employed as validation set 2. Two images from each lesion were tested, and trials were categorized as correct if the judgement (≥ 1 image) was correct. We used precision (Pr), recall rate (Rc), F1 score (F1), and the area under the receiver operating characteristic curve (AUROC) as the performance metrics of the DTL model with validation set 2.

**Results:**

The S5 model achieved the best fit for the data. The Pr, Rc, F1 and AUROC of S5 were 0.90, 0.90, 0.90, and 0.86, respectively, for Category 4. The proportions of lesions downgraded by S5 were 90.73%, 84.76%, and 80.19% for categories 4 A, 4B, and 4 C, respectively. The overall proportion of BI-RADS 4 lesions downgraded by S5 was 85.91%. There was no significant difference between the classification results of the S5 model and pathological diagnosis (*P* = 0.110).

**Conclusion:**

The S5 model we proposed here can be used as an effective approach for reducing the number of unnecessary biopsies that residents need to conduct for mammographic BI-RADS 4 lesions and may have other important clinical uses.

**Supplementary Information:**

The online version contains supplementary material available at 10.1186/s12880-023-01023-4.

## Background

Recent data demonstrate that female breast cancer accounted for approximately 11.7% of the total number of new cancer cases worldwide in 2020 and has surpassed lung cancer as the most commonly diagnosed cancer, with an estimated 2.3 million new cases [1]. Mammography is an important imaging tool for breast cancer screening and diagnosis in clinical practice. In the fifth edition of the American College of Radiology’s Breast Imaging Reporting and Data System (BI-RADS) updated in 2013, BI-RADS category 4 lesions have a high likelihood of being diagnosed as cancer (2–95%) and can be further divided into three subcategories: 4 A (malignancy probability:>2–10%), 4B (malignancy probability: >10–50%), and 4 C (malignancy probability: >50%–<95%) [2]. The fifth edition of the BI-RADS recommends that “biopsy should be performed in the absence of clinical contraindication” for category 4 lesions [3]. Microcalcifications play an important role in the subclassification of breast lesions; however, their judgement differs widely among physicians, particularly among residents in training. This practice may lead to unnecessary biopsy of a large percentage of BI-RADS category 4 lesions and impose a certain degree of economic burden and additional pressure on the patient [4]. Thus, there is significant room for improvement in reducing unnecessary biopsies [5], and a new method that has higher specificity than classical methods is required to address this issue.

Deep transfer learning (DTL) is an effective strategy for adapting a pretrained neural network to a new domain. In contrast to traditional conventional visual image analysis, effective image features are automatically learned and extracted by DTL. Active research has been conducted on the application of DTL in terms of disease detection [6], classification [7–9] and evaluation of the response to different treatments [10, 11].

Montaha et al. [12] proposed a BreastNet18 model based on the fine-tuned VGG16 for diagnosing breast cancer from enhanced mammography images. The results showed that the BreastNet18 model reached a training accuracy of 96.72%, a validating accuracy of 97.91%, and a test accuracy of 98.02%. It has been demonstrated in this study that a high correct classification of breast cancer was achieved when dealing with a limited number of complex medical images.

In another study, Mahmood et al. [13] applied ConvNet + SVM model to differentiate breast masses in mammography images, and the model performed best with a discriminative training accuracy of 97.7%, contrary to this, VGG16 method yielded 90.2%, 93.5% for VGG19, 63.4% for GoogLeNet, 82.9% for MobileNetV2, 75.1% for ResNet50, and 72.9% for DenseNet121. They concluded that the proposed model’s improvement and validation are appropriated in conventional pathological practices that conceivably reduce the pathologist’s strain in predicting clinical outcomes by analyzing patients’ mammography images. Nevertheless, advanced pretraining strategies are important for deep learning-based classification tasks [14].

In this study, we constructed a DTL model for the identification of breast lesions based on Inception V3, and different fine-tuning strategies were used to improve its performance. We focused on the potential value of the fine-tuned model in reducing the number of unnecessary biopsies that residents must perform for mammographic BI-RADS 4 lesions.

## Methods

### Mammography

Digital mammographic examinations were performed using a Senographe 2000D system (GE Healthcare). Automatic exposure mode was chosen, and the tube voltage was set to 34 kV. Standard craniocaudal (CC) and mediolateral oblique (MLO) positions were assumed by all patients; all glandular breast tissue was included, and bilateral symmetry was considered. The institutional review board approved this retrospective study and waived the requirement for informed consent. We confirm that all methods were performed in accordance with the relevant guidelines and regulations.

### Study population

The study population consisted of patients admitted to our hospital between January 1, 2016, and June 30, 2021. All patients had complete pathological and mammography data. A total of 1980 patients with breast lesions were included, including 1473 benign lesions (185 women with bilateral breast lesions), and 692 malignant lesions collected and confirmed by clinical pathology or biopsy. The data are summarized in Table 1. Patients in the malignant group (55.39 ± 11.57 years) had a higher mean age than those in the benign group (41.64 ± 10.78 years) (*P* < 0.05).

**Table 1:** Clinical information of the patients

Pathological diagnosis	Lesions	Percent (%)	Age (years)	
Malignant lesions				55.39 ± 11.57
Invasive ductal carcinoma	587	84.83		
Intraductal carcinoma	68	9.83		
Invasive lobular carcinoma	13	1.88		
Mucinous carcinoma	15	2.17		
Lymphoma	3	0.43		
Papillary carcinoma	6	0.87		
Total	692	100.00		
Benign lesions				41.64 ± 10.78
Cyst	144	9.78		
Adenosis	228	15.48		
Fibroadenoma	940	63.82		
Chronic inflammation	61	4.14		
Intraductal papilloma	88	5.97		
Lobular tumor	12	0.08		
Total	1473	100.00		

### Data preparation

#### Training and testing sets

Ultimately, 4330 images from 1980 patients were obtained, including 2946 (68.04%) benign images (and 1384 (31.96%) malignant images. These images were randomly divided into the training set (2358 benign and 1108 malignant), testing set (294 benign and 138 malignant), and validation set 1 (294 benign and 138 malignant) at a ratio of 8:1:1. Data augmentation (rotation range 60°, shear range 0.2, zoom range 0.2, horizontal flip, vertical flip) was performed to increase the number of images in the training and testing sets before the beginning of training.

#### Validation set 2

Another 362 patients, diagnosed with BI-RADS 4 category lesions by 5 residents, were used as the validation set. Of these, 151 patients were classified into category 4 A, 105 into category 4B, and 106 into category 4 C. All residents were trained according to the fifth edition of the BI-RADS mammography criteria. All selected lesions were mass-like and were single lesions (lesions in either the left or right breast). Validation Set 2 was used to test the robustness of the DTL model. Two images (CC and MLO) for each lesion were used, and if one of the two images was classified correctly, we considered the patient to be classified correctly.

### DTL diagram

**Fig. 1:**
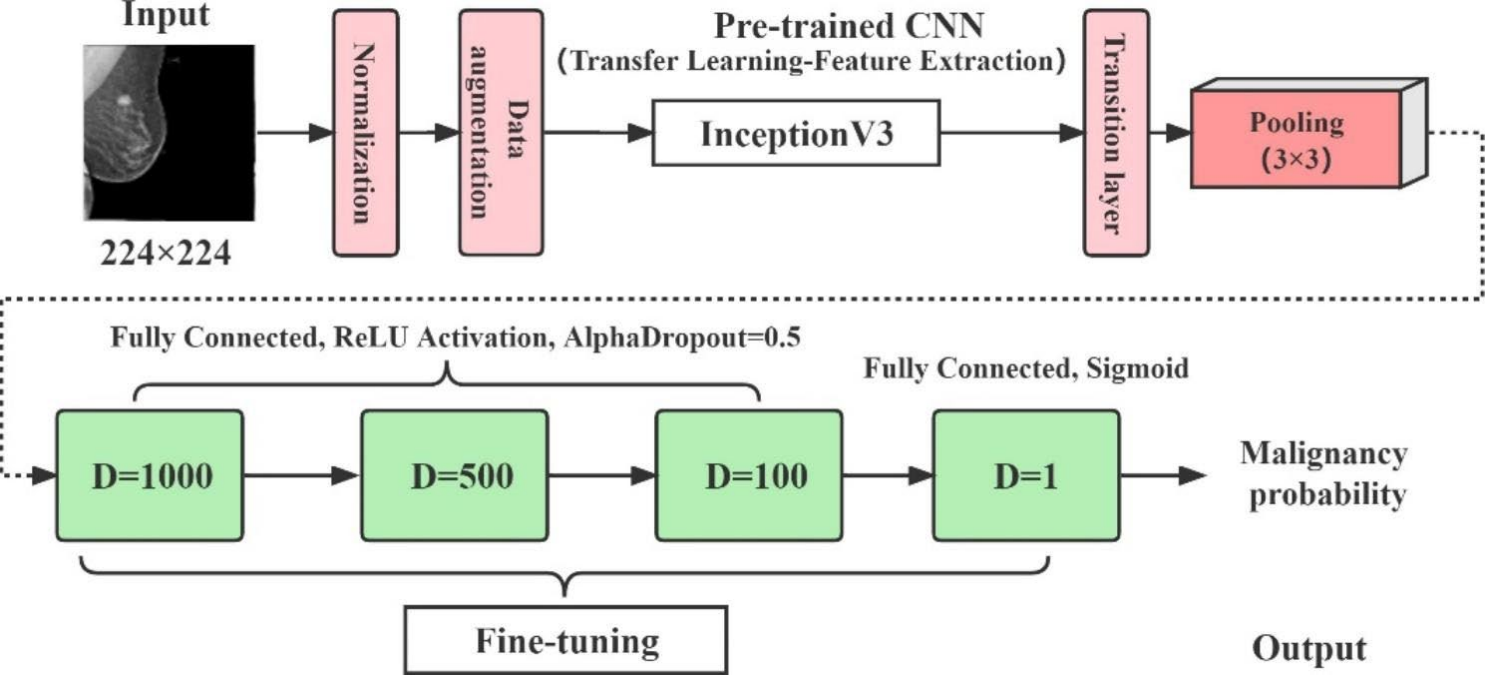
DTL diagram. The format of the input images was BMP. The process is divided into three parts: image neural network feature extraction, model training and testing, and model validation. The selected optimizer was the “Adam” optimizer, learning rate was 0.0001

The computer used to run the model contained an Intel(R) Core (TM) i7-10700 F CPU with an ASUS GeForce RTX 2060 6G GPU. The Python programming language (Python Software Foundation, version 3.6) was used for our analysis, and Keras (version 2.2.4) with TensorFlow (version 2.0) was used in the backend. All other processes were turned off while the program was running. The DTL model based on a pretrained deep learning network model (Inception V3, imported from Keras) is shown in Fig. 1. The training and testing processes of the DTL model were recorded using a computer.

We used binary cross-entropy as our loss function and the stochastic gradient descent (SGD) optimizer to minimize the loss, with an epoch parameter of 1000. In addition, the learning rate was 0.001, and the activation functions were ReLU and sigmoid, defined in Eqs. 1 and 2:

1 $$Relu\left(x\right)=f\left(x\right)=\left\{\begin{array}{c}max(0,x), x\ge 0\\ 0, x<0\end{array}\right.$$

2 $$\text{S}\text{i}\text{g}\text{m}\text{o}\text{i}\text{d}\left(x\right)=f\left(x\right)=\frac{1}{1+{e}^{-x}}$$

The data analysis process is divided into three parts: image network feature extraction, data training and testing, and validation of the DTL model.

### Fine-tuning strategy

**Fig. 2:**
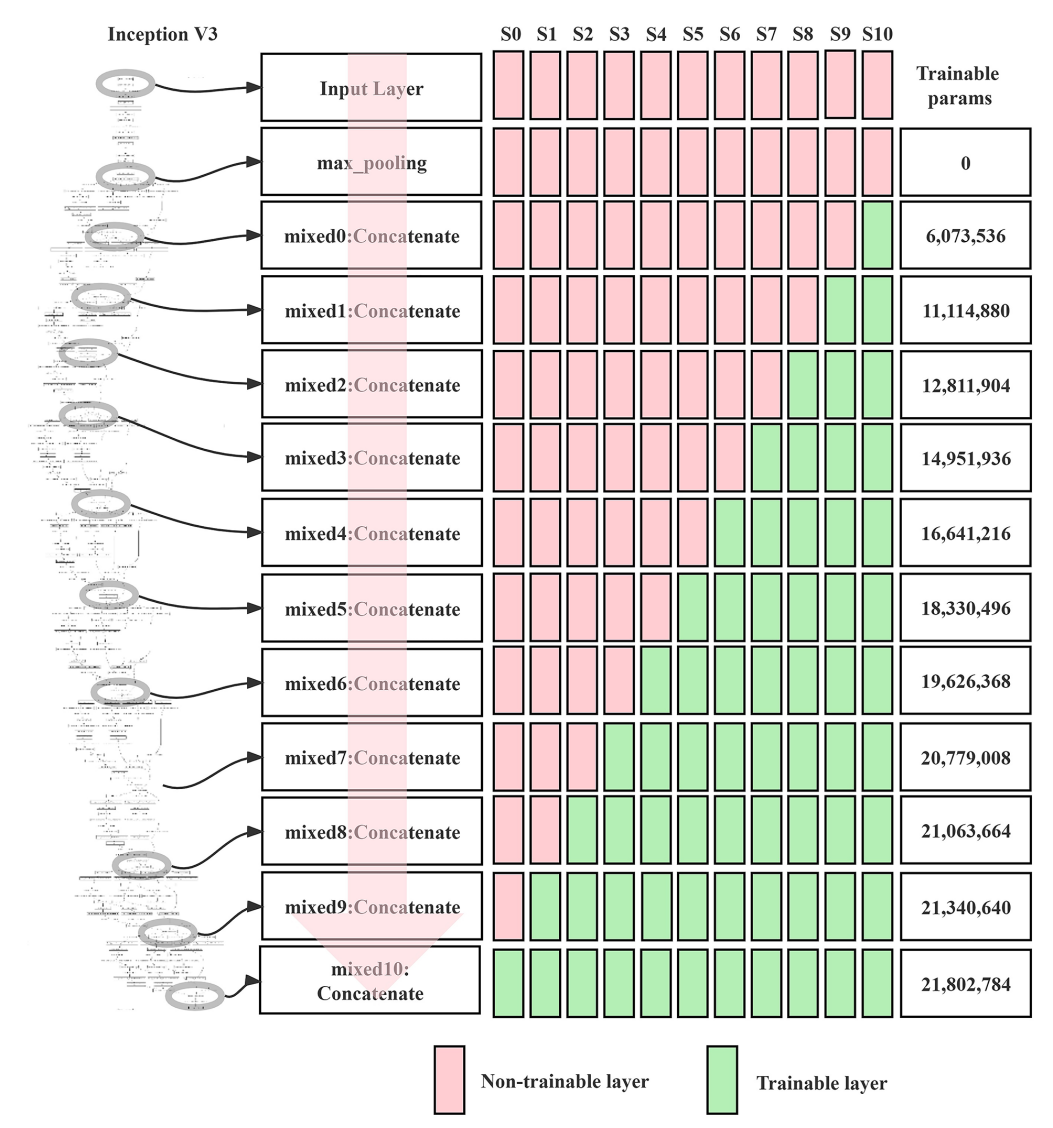
Schematic diagram of fine-tuning strategies for Inception V3. There were eleven fine-tuning strategies in total. Note: Trainable params, the number of trainable parameters. Trainable layer: activated layers of the neural network. Non-trainable layer: frozen layers

**Fig. 3:**
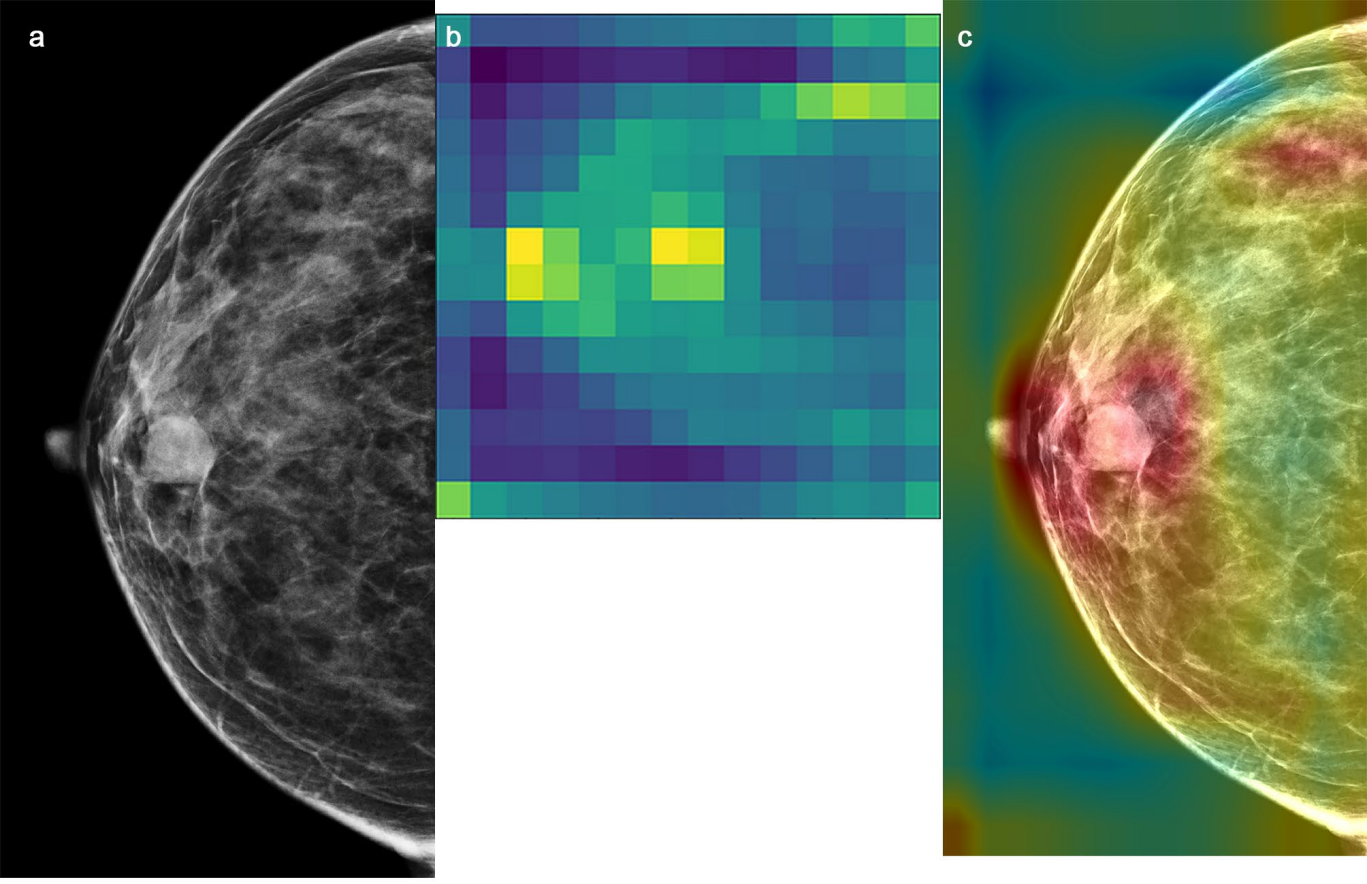
Class activation heatmap for a malignant lesion. **a**: input mammographic image; the white arrow shows a benign breast lesion. **b:** Heatmap of **(a)c**: Fusion image of **a** and **(b)** The large intensity of activation in the breast lesion reinforcement region is evident from the heatmap, which may reflect the difference between benign and malignant breast lesions identified by the convolutional neural network. The lesion was pathologically confirmed as a fibroadenoma

We fine-tuned a total of 11 layers, they were Mixed 0 ~ Mixed 10 layers, activated parameters corresponding to these layers are from 6 to 536 to 21 802 784. We sought to improve the performance of the Inception V3 model by devising 11 preset fine-tuning strategies. The parameters of the neural network were activated and participated in the model training process, whereas the parameters of the layers that were kept frozen were not involved in training the model (Fig. 2). We selected the parameter convergence and generalization capacity as the primary outcome measures for the DTL models. A visualization of the activation heatmap in the DTL model is shown in Fig. 3. Activation heatmap were made as described in reference [15].

### Network performance evaluation

To compare the performance of each model, five performance indices were calculated as metrics in this study: accuracy (Ac), precision (Pr), recall rate (Rc), F1 score (F1), and area under the receiver operating characteristic curve (AUROC).

3 $$\text{A}\text{c}=\frac{\text{T}\text{P}+\text{T}\text{N}}{\text{T}\text{P}+\text{T}\text{N}+\text{F}\text{P}+\text{F}\text{N}}$$

4 $$\text{P}\text{r}=\frac{\text{T}\text{P}}{\text{T}\text{P}+\text{F}\text{P}}$$

5 $$\text{R}\text{c}=\frac{\text{T}\text{P}}{\text{T}\text{P}+\text{F}\text{N}}$$

6 $$\text{F}1=\frac{2\times \text{A}\text{c}\times \text{R}\text{c}}{\text{A}\text{c}+\text{R}\text{c}}$$

In our study, positive and negative cases were assigned to the malignant and benign groups, respectively. Hence, true positive (TP) and true negative (TN) represent the numbers of correctly diagnosed malignant and benign lesions, respectively, while False positive (FP) and false negative (FN) indicate the number of incorrectly diagnosed malignant lesions and benign lesions, respectively.

### Statistical analysis

Statistical analysis was performed using SPSS 23.0 statistical software (IBM). The age of the patients is represented as mean ± standard deviation ($\stackrel{-}{x}$ ± s). One-way analysis of variance (ANOVA) was used to analyze the variance between the two groups. We compared frequencies by *Chi-square test.* Statistical significance was set at *P* < 0.05.

## Results

### Results for the training and testing sets

The results showed that the accuracy of the training set reached 100.00% for all fine-tuning strategies after 1000 epochs; however, only strategy 5 achieved the best test accuracy of 82.16%. As the number of epochs for the training set increased, the training loss value decreased for all fine-tuning strategies. During the testing process, the test loss tended to increase for all fine-tuning strategies except S5. This result suggests that of the fine-tuning models, only S5 converged. We found that the test accuracy (82.16%) of S5 was the highest of all fine-tuning strategies, further illustrating that the S5 model was a better fit than the other models. Next, 10-fold cross-validation was employed to evaluate the S5 model. We were surprised that the sizes of all H5 files saved by the 11 fine-tuning strategies were the same. Furthermore,

as the number of parameters participating in the model training process increased, the time consumption also tended to increase.

### Validation results for the fine-tuning strategies

The lesions of 362 patients diagnosed with BI-RADS 4 were subjected to histopathology; of these, 294 were diagnosed with benign tumours, and 68 were diagnosed with malignant lesions. The overall malignancy rate (i.e., positive predictive value (PPV) of histopathology) was 18.78%; according to subcategory, the rates were 9.93% (15/151) for category 4 A, 21.90% (23/105) for category 4B and 28.30% (30/106) for category 4 C. The proportion of lesions downgraded by the S5 model was 85.91% for category 4 and 90.73%, 84.76% and 80.19% for categories 4 A, 4B and 4 C, respectively. There was a statistically significant difference in the proportions of downgraded BI-RADS 4 A-C lesions; the proportion was significantly lower for 4 A than for 4B and 4 C (*P* < 0.05). There was no statistically significant difference in the BI-RADS 4 lesion classification between S5 and histopathology (*P* = 0.110). There was no statistically significant difference in the proportions of downgraded lesions among the five residents (*P* = 0.110). There was a statistically significant difference in the PPV for histopathology for 4 A, 4B and 4 C lesions; the PPV for histopathology was significantly lower for 4 A than for 4B and 4 C lesions (*P* < 0.05). Further details are provided in Table 2.

**Table 2:** Downgraded lesions by the DTL model and histopathology results in the validation set

R	N1				N2				Histologically benign				Histologically malignant			
	4 A	4B	4 C		4 A	4B	4 C		4 A	4B	4 C		4 A	4B	4 C	
R1	29	22	19		27	19	16	DTL benign	26	18	14		1	1	2	
								DTL malignant	1	1	0		1	2	3	
R2	32	20	22		29	16	17	DTL benign	28	15	15		1	1	2	
								DTL malignant	1	0	1		2	4	4	
R3	29	19	20		26	17	16	DTL benign	25	15	14		1	2	2	
								DTL malignant	1	0	1		2	2	3	
R4	31	24	21		27	21	17	DTL benign	26	19	14		1	2	3	
								DTL malignant	1	0	0		3	3	4	
R5	30	20	24		28	16	19	DTL benign	27	14	16		1	2	3	
								DTL malignant	0	0	1		2	4	4	
Total	151	105	106		137	89	85		136	82	76		15	23	30	

Note: R, resident; N1, number of BI-RADS 4 lesions diagnosed by residents; N2, number of downgraded lesions according to the DTL model

The classification report of the S5 model in validation set 2 is presented in Table 3. The Pr, Rc, F1, and AUROC values of the S5 model with validation set 2 were 0.90, 0.90, 0.90, and 0.86, respectively. The AUROCs of the DTL model for 4 A, 4B, and 4 C lesions were 0.80, 0.89, and 0.85, respectively.

**Table 3:** Classification report of the DTL model in the validation set

Group	precision					recall					F1 score					support images				
	4 A	4B	4 C	all		4 A	4B	4 C	all		4 A	4B	4 C	all		4 A	4B	4 C	all	
Group1	0.96	0.90	0.87	0.92		0.96	0.98	0.95	0.96		0.96	0.94	0.91	0.94		276	183	171	630	
Group2	0.65	0.88	0.83	0.99		0.65	0.63	0.61	0.63		0.65	0.73	0.71	0.70		26	27	41	94	
Avg/total	0.93	0.90	0.86	0.90		0.93	0.90	0.86	0.90		0.93	0.89	0.86	0.90		302	210	212	724	

Note: Group 1, benign group; Group 2, malignant group; Avg, average

## Discussion

A standard procedure has been developed to manage breast lesions in the BI-RADS atlas, which mandates providing a BI-RADS assessment category based on the most suspicious imaging features [3]. BI-RADS assessment categories inform clinicians about the possibility of malignancy and how to manage breast lesions, and it is important for radiology residents to understand the lexicon so that they can effectively communicate breast imaging findings depicted on mammography [16].

Our data showed that the PPV for histopathology for 4 A, 4B, and 4 C lesions was 9.93% (15 of 151) 21.90% (23 of 105) and 28.30% (30 of 106), respectively. The PPVs for 4 A and 4B were within the specified BI-RADS malignancy ranges, but the PPV for 4 C lesions was much lower than the corresponding malignancy range (50–95%), which could be due to the limited amount of data. In clinical practice, the diagnosis of BI-RADS category 4 can be influenced by the clinical experience of the radiologist, particularly that of residents, who limited experience makes them more inclined to over diagnose on the initial mammographic images. The overall PPV for histopathology for validation set 2 (362 patients) was 18.78%, which is lower than the values reported in the literature [17], indicating that the residents tended to over diagnose the lesions in validation set 2. A method for downgrading BI-RADS category 4 lesions could further reduce the number of unnecessary biopsies for benign lesions as well as the financial burden on patients and medical resources can also be saved.

Continuous improvements in computer hardware and deep learning algorithms have helped AI make significant achievements in the field of medical auxiliary diagnosis. The transfer learning method is a validated tool for adapting a pretrained neural network to a new domain, particularly one with a smaller dataset. For example, in the field of medical image classification for rare and emerging diseases. The study of Zhou et al. [18] showed that Inception V3 model can effectively predict clinically negative axillary lymph node metastasis. A recent study assessed the diagnostic performance of six deep convolutional neural networks in classifying breast microcalcifcation in screening mammograms, result showed that the ResNet-101 model yielded a higher Ac (81.54%) than that of Inception V3(77.69%) [19]. Alom et al. [20] adopted the inception recurrent residual convolutional neural network model to classify the pathological picture of breast cancer and achieved a classification accuracy of 97.51%. Another study showed that the application of Inception V3 to meningioma apparent diffusion coefficient (ADC) maps provided high diagnostic accuracy results, with an AUROC of 0.94 [21]. However, some studies reported different findings, with Inception V3 achieving relatively low accuracies. For example, in the diagnosis of retinitis pigmentosa, the accuracy was only 68.00%, which was lower than that of Xception (80.00%) and Inception Resnet V2 (75.00%) [22].

These data suggest that the results of Inception V3 can differ for different datasets. The results of another article gave us confidence. Zhang et al. [23] adopted InceptionV3 model to investigate its diagnostic efficiency in breast cancer in ultrasound images, result showed the AUC (0.913) of the InceptionV3 model was larger than that (0.846) obtained by sonographers. Our study showed that the accuracy of the default Inception V3 model (S0) was only 74.05% in our dataset. After fine-tuning using S5, the model achieved an accuracy of 82.16%.This indicates that in our dataset, the training parameters of Inception V3 were not optimal, and use of only some of the parameters yielded the best results. This is inconsistent with the results reported by Singh et al. [24], who found that in the detection of critical enteric feeding tube malpositioning on radiography, the pretrained Inception V3, which had an AUROC of 0.87, performed significantly better than the untrained Inception V3, with an AUROC of 0.60. Hence, there is room to further optimize and improve the performance of Inception V3, but this requires more training data and the exploration of new and more feasible fine-tuning methods. Of course, a quality control program based on machine learning algorithms is also important [25]. We expect to conduct such research in the future. Wang et al. [26] use a modified Inception-v3 architecture to assist radiologists in breast cancer classification in automated breast ultrasound imaging, their method achieved an AUROC value of 0.95, for which the sensitivity and specificity were 88.60% and 87.6%, respectively.

One study showed that deep learning-based mammogram calcification detection systems show high sensitivity and stability, which may help to reduce the miss rate for calcifications (especially in suspicious images) [27]. The results of the present study showed that the proportion of lesions downgraded by the S5 model was 85.91% for category 4 and 90.73%, 84.76% and 80.19% for categories 4 A, 4B and 4 C, respectively. There was no statistically significant difference in the BI-RADS 4 lesion classification between the S5 model and histopathology, which illustrates that the S5 model has the potential to improve the accuracy of mammography-based breast disease diagnosis in clinical settings. This is consistent with the results reported by Zhao et al. [28], who showed that with the help of deep learning software, the specificity, overall diagnostic performance, and interobserver agreement of the residents greatly improved, suggesting that the software can be used as an adjunctive tool for residents, downgrading 4a lesions to possibly benign and reducing unnecessary biopsies.

Our data also showed that there was a statistically significant difference in the proportions of downgraded BI-RADS 4 A-C lesions; specifically, there were significantly fewer downgraded 4 A lesions (90.73%) than 4 B (84.76%) and 4 C lesions (80.19%). This illustrates that for category 4 A, the likelihood of overdiagnosis is higher than that for 4B and 4 C in clinical practice. There are numbers of possible reasons for this observation. Architectural distortion and calcification morphology are the most significantly associated findings with the use of category 4 subdivisions [3, 17]. There are two points to note for category 4 A: ①Similar to adenomas, this category may include a partially (< 75%) circumscribed solid mass. ②Coarse heterogeneous microcalcifications have a 7% likelihood of malignancy. Fine pleomorphic and amorphous calcifications have a 13–29% likelihood of malignancy and are typical signs of BI-RADS 4B. Fine linear and branching calcifications have a 53% likelihood of malignancy and support the diagnosis of 4 C. Therefore, we believe that identification of the signs of 4 A is relatively difficult for residents to master, while the signs of 4B and 4 C are relatively easy to grasp.

Several limitations of this pilot study must be acknowledged. First, the number of images in the training set was relatively small, particularly due to the lack of rare lesions. Our training dataset also may not represent the entire population of breast disease patients, which may impact the accuracy of the DTL model. Therefore, further analysis with additional data is necessary to fully test the robustness of the DTL model. Second, during routine diagnostic procedures, clinical evaluation, breast ultrasound, and magnetic resonance imaging are performed in addition to mammography. However, only static mammographic images were used in our study. Third, the high performance achieved by our proposed model is based on the premise of high-quality mammographic images. In clinical practice, poor-quality images from other hospitals may reduce the performance of the DTL model. Therefore, high-quality mammographic images obtained using standard procedures are highly warranted. A future study might require multicentre collaboration to obtain a sufficiently large series of data to train and test the neural network [29]. In terms of the time consumption, the training time increased with the number of trainable parameters. However, this time consumption is acceptable.

## Conclusion

An Inception V3 model with the S5 strategy can be used as an effective approach for downgrading mammographic BI-RADS 4 lesions, enabling the avoidance of artificial subjective factors and reducing the number of unnecessary biopsies. However, this DTL model may need further refinement before it can be used in clinical practice. In this study, we demonstrated its potential value for future clinical applications.

## Electronic supplementary material

Below is the link to the electronic supplementary material.

Supplementary Material 1

## Data Availability

The datasets generated and/or analysed during the current study are not publicly available because the datasets are under continuous development and refinement but are available from the corresponding author upon reasonable request.

## Abbreviations

AUROC: area under the receiver operating characteristic curve
CNN: convolutional neural network
ROC: receiver operating characteristic
DTL: deep transfer learning
BI-RADS: Breast Imaging Reporting and Data System
CC: craniocaudal
MLO: mediolateral oblique
PPV: positive predictive value
