# Supplementary material for: Reducing the number of unnecessary biopsies for mammographic BI-RADS 4 lesions through a deep transfer learning method
Source: BMC Med Imaging. 2023 Jun 13;23:82. doi: 10.1186/s12880-023-01023-4 (PMC10265786; doi:10.1186/s12880-023-01023-4)
Supplement: Supplementary file 1 — Supplementary Material 1 [file 12880_2023_1023_MOESM1_ESM.pdf]

Code used in this article

```
from tensorflow.keras.callbacks import
ReduceLROnPlateau,ModelCheckpoint
from tensorflow.keras.preprocessing.image import ImageDataGenerator
from sklearn.metrics import confusion_matrix
import tensorflow as tf
import matplotlib.pyplot as plt
from tensorflow import keras
from PIL import Image
get_ipython().run_line_magic('matplotlib', 'inline')
import sklearn
import pandas as pd
import sys
import numpy as np
import itertools
from sklearn.metrics import roc_curve,auc
import os
import matplotlib.pyplot as mp
import sklearn.naive_bayes as nb
import sklearn.model_selection as ms
import sklearn.metrics as sm
from sklearn.metrics import recall_score
from sklearn.model_selection import train_test_split
from sklearn import metrics
import time

print(tf.__version__)
print(sys.version_info)
for module in np,sklearn, tf,pd, keras:
    print(module.__name__, module.__version__)

if not os.path.exists("save_weights"):
    os.makedirs("save_weights")

im_height = 224
im_width = 224
batch_size =64
epochs = 500
image_path =""
train_dir = image_path + "train"
validation_dir = image_path + "valid"
test_dir = image_path + "test"
```

```
train_image_generator = ImageDataGenerator(rescale=1./255,
                                    shear_range=0.2,
                                    zoom_range=0.2,
                                    horizontal_flip=True)
validation_image_generator = ImageDataGenerator(rescale=1./255)
test_image_generator = ImageDataGenerator(rescale=1./255)


train_data_gen =
train_image_generator.flow_from_directory(directory=train_dir,

batch_size=batch_size,
                                                  shuffle=False,

target_size=(im_height, im_width),

class_mode="sparse",classes=["benign","malignant"])
total_train = train_data_gen.n

val_data_gen =
validation_image_generator.flow_from_directory(directory=validation_d
ir,

batch_size=batch_size,
                                                    shuffle=False,

target_size=(im_height, im_width),

class_mode='sparse',classes=["benign","malignant"])

total_val = val_data_gen.n

test_data_gen =
test_image_generator.flow_from_directory(directory=test_dir,
                                                batch_size=batch_size,
                                                shuffle=False,

target_size=(im_height, im_width),

class_mode='sparse',classes=["benign","malignant"])

total_test = test_data_gen.n
```

```
covn_base = tf.keras.applications.InceptionV3(weights='imagenet',
include_top=False,input_shape=(im_height,im_width,3),pooling='avg')
covn_base.trainable = False

def extract_feature(data,model,path):
   results=[]
   count=0
   for s in data:
      count+=1
      vector,label=s
      vector=model.predict(vector)[0].tolist()
      print(count,data.n)
      label=label.tolist()
      results.append(str([vector,label]))
      if count==data.n:
         break
   with open(path,"w") as f:
      f.writelines("\n".join(results))

start = time.clock()
model = tf.keras.Sequential()
model.add(covn_base)
#model.compile(optimizer='adam',loss='categorical_crossentropy',metri
cs=['accuracy'])
model.compile(optimizer=tf.keras.optimizers.Adam(learning_rate=0.0001)
,loss='binary_crossentropy',metrics=['accuracy'])
extract_feature(train_data_gen,model,"train")
extract_feature(test_data_gen,model,"test")
extract_feature(val_data_gen,model,"valid")

model.layers
model.summary()


def read_data(path):
   with open(path) as f:
      lines=f.readlines()
   x,y=zip(*[eval(line.strip()) for line in lines])
   x=np.array(x)
   y=np.array(y)
   return x,y

x_train,y_train=read_data("train")    #("feature/train")
```

```
x_test,y_test=read_data("test")   #("feature/test")
print(x_train.shape, y_train.shape)
print(x_train.shape)
print(x_test.shape, y_test.shape)
print(x_test.shape)
print(x_test.shape)
print(y_test.shape)


im_height = 224
im_width = 224
covn_base = tf.keras.applications.InceptionV3(weights='imagenet',
include_top=False,input_shape=(im_height,im_width,3),pooling='avg')
dnn1=tf.keras.layers.Dense(1000, activation='relu')
dnn2=tf.keras.layers.Dense(500, activation='relu')
dnn3=tf.keras.layers.Dense(100, activation='relu')
model.add(tf.keras.layers.Flatten())
model.add(keras.layers.AlphaDropout(rate=0.5))
dnn4=tf.keras.layers.Dense(1, activation='sigmoid')#softmax
model = tf.keras.Sequential()
model.add(dnn1)
model.add(dnn2)
model.add(dnn3)
model.add(dnn4)
model.compile(optimizer=tf.keras.optimizers.Adam(learning_rate=0.0001)
,loss='binary_crossentropy',metrics=['accuracy'])

logdir =os.path.join("callbacks")
if not os.path.exists(logdir):
   os.mkdir(logdir)
output_model_file = os.path.join(logdir,
                            "InceptionV3.h5")

callbacks = [keras.callbacks.TensorBoard(logdir),
   keras.callbacks.ModelCheckpoint(output_model_file,
                              save_best_only = True),
   keras.callbacks.EarlyStopping(patience=5, min_delta=1e-3),]

history
=model.fit(x=x_train,y=y_train,epochs=100,batch_size=128,validation_d
ata=(x_test,y_test),class_weight='auto')
print(time.clock() - start)

model2 = tf.keras.Sequential()
```

```
model2.add(covn_base)
model2.add(dnn1)
model2.add(dnn2)
model2.add(dnn3)
model2.add(dnn4)
model2.save("")
model.save_weights('',save_format='tf')

model.summary()
model.layers
model.evaluate(x_test,y_test,verbose=0)

def plot_learning_curves(history):
    pd.DataFrame(history.history).plot(figsize=(7, 5))
    plt.ylabel('Accuracy')
    plt.xlabel('Epoch')
    plt.grid(True)
    plt.gca().set_ylim(0, 1.05)
    plt.show()
plot_learning_curves(history)


im_height=224
im_width=224
batch_size = 64
epochs =500
test_dir = ""
test_image_generator = ImageDataGenerator(rescale=1./255)
test_data_gen =
test_image_generator.flow_from_directory(directory=test_dir,
                                                 batch_size=batch_size,
                                                 shuffle=False,

target_size=(im_height, im_width),

class_mode='sparse',classes=["benign","malignant"])

total_test = test_data_gen.n
covn_base =tf.keras.applications.InceptionV3(weights='imagenet',
include_top=False,input_shape=(im_height,im_width,3),pooling='avg')
covn_base.trainable = False

import sklearn.metrics as sm
from sklearn import metrics
```

```
def predict(data,model):
   y_true=[]
   y_predict=[]
   count=0
   for s in data:
      count+=1

      img,label=s

      pred_y=model.predict(img)[0].tolist()[0]
      label=label.tolist()[0]
      y_true.append(label)
      y_predict.append(pred_y)
      label="" if label==1 else ""
      print(count,":"+str(round(pred_y,3)),":"+label)
      if count>=data.n:
         break
   return y_true,y_predict

model=tf.keras.models.load_model("InceptionV3.h5")
y_true,y_predict=predict(test_data_gen,model)
auc = metrics.roc_auc_score(y_true,y_predict)
print("auc",auc)
predict_label=[0 if s<0.5 else 1 for s in y_predict]


print("auc",auc)
m = sm.confusion_matrix(y_true,predict_label)
print("confusion_matrix",m,sep='\n')

r = sm.classification_report(predict_label,y_true)
print(': ', r, sep='\n')

mp.xticks([0,1])
mp.yticks([0,1])
plt.ylabel('Predicted label')
plt.xlabel('True label')
mp.imshow(m, cmap='Blues')
mp.show()

from sklearn.metrics import roc_curve,auc
fpr,tpr,thresholds = roc_curve(y_true,predict_label)
def plot_roc_curve(fpr, tpr):
```

```
    plt.figure(figsize=(7, 5))
    lw = 3
    roc_auc = auc(fpr,tpr)
    plt.plot(fpr, tpr, color='g',lw=lw,
            label='InceptionV3 ROC curve (area = %0.2f)' % roc_auc)
    plt.plot([0,1], [0,1], color='navy', lw=lw, linestyle='--')
    plt.xlim([0, 1.00])
    plt.ylim([0, 1.05])
    plt.xlabel('False Positive Rate')
    plt.ylabel('True Positive Rate')
    plt.title('Receiver operating characteristic example')
    plt.legend(loc="lower right")
    plt.show()
plot_roc_curve(fpr, tpr)
```
